# Low Oxygen Tension Maintains Multipotency, Whereas Normoxia Increases Differentiation of Mouse Bone Marrow Stromal Cells

**DOI:** 10.3390/ijms14012119

**Published:** 2013-01-22

**Authors:** Ina Berniakovich, Marco Giorgio

**Affiliations:** Department of Experimental Oncology, European Institute of Oncology, Via Adamello16, 20139 Milan, Italy

**Keywords:** hypoxia, mesenchymal stem cells, differentiation, oxygen, bone marrow stromal cells

## Abstract

Optimization of mesenchymal stem cells (MSC) culture conditions is of great importance for their more successful application in regenerative medicine. O_2_ regulates various aspects of cellular biology and, *in vivo*, MSC are exposed to different O_2_ concentrations spanning from very low tension in the bone marrow niche, to higher amounts in wounds. In our present work, we isolated mouse bone marrow stromal cells (BMSC) and showed that they contained a population meeting requirements for MSC definition. In order to establish the effect of low O_2_ on cellular properties, we examined BSMC cultured under hypoxic (3% O_2_) conditions. Our results demonstrate that 3% O_2_ augmented proliferation of BMSC, as well as the formation of colonies in the colony-forming unit assay (CFU-A), the percentage of quiescent cells, and the expression of stemness markers Rex-1 and Oct-4, thereby suggesting an increase in the stemness of culture when exposed to hypoxia. In contrast, intrinsic differentiation processes were inhibited by 3% O_2_. Overall yield of differentiation was dependent on the adjustment of O_2_ tension to the specific stage of BMSC culture. Thus, we established a strategy for efficient BMSC *in vitro* differentiation using an initial phase of cell propagation at 3% O_2_, followed by differentiation stage at 21% O_2_. We also demonstrated that 3% O_2_ affected BMSC differentiation in p53 and reactive oxygen species (ROS) independent pathways. Our findings can significantly contribute to the obtaining of high-quality MSC for effective cell therapy.

## 1. Introduction

The multi-differentiation potential of MSC makes them attractive as potential tool for regenerative medicine. The conditions for their isolation, propagation and differentiation involve changes in O_2_ in the environment and exposure to oxidative stress. In different cell types, O_2_ tension has a clear effect on stem cell properties such as stemness, division, differentiation and recruitment to organs [1–5]. Investigating MSC response to O_2_ variations may not only reveal insights in stem cell biology, but also improve their clinical applications.

So far, the majority of research has been focused on human MSC (hMSC) since they are well defined, represent a homogeneous population, and show a high *in vitro* proliferation rate [6]. MSC isolated from mouse (mMSC) can be used as a helpful experimental tool to investigate genetic and environmental factors to ameliorate MSC handling. Studies on murine MSC are restricted by a high heterogeneity of primary cultures, lack of CD set for specific MSC isolation, and decline in proliferation with time [7,8]. The first important step towards improvement of the mMSC model is the identification of the best *in vitro* culture conditions. Standard cell culture is routinely performed at 21% O_2_ tension which is hyperoxic compared with that of natural cell niche (for review see [9]). Therefore, modification of O_2_ tension during isolation and cultivation of mMSC would presumably affect their growth and differentiation potential.

The effect of low O_2_ tension on the cellular growth was well demonstrated in hMSC, where it induced proliferation [10,11]. In mice, a switch in culture from 21% to 8% O_2_ also stimulated proliferation and increased the number of cells in S-G2/M phases in the whole population of BMSC cells [12]. However, the effect of low O_2_ on the pure population of mMSC was not shown.

The reported effects of low O_2_ tension on MSC differentiation are disputable. In humans, both inhibitory and enhancing effects of hypoxia on osteocytic differentiation were reported [10,11,13,14]. Culture under hypoxic conditions was beneficial for the osteocytic differentiation of rat MSC [15], whereas it decreased osteocytic differentiation of mMSC isolated from adipose tissue [16]. An inhibitory effect of low O_2_ tension on adipocytic differentiation was observed in hMSC by Fehrer [11]. In contrast, another study demonstrated that, in hMSC, very low oxygen augmented lipogenesis without impact on the expression of markers of adipocytic differentiation [17]. In mice, exposure of MSC isolated from adipose tissue enhanced their adipogenic differentiation potential [18].

Controversies in the aforementioned observations can be explained by the fact that O_2_ simultaneously regulates multiple cellular processes. Therefore in order to draw a clear conclusion on O_2_’s role, one has to take into account that the final cell fate is determined by the collective effects of O_2_; the growth of cell culture, for instance, depends on the balance between the proliferation rate and cell survival, and the differentiation yield is determined by both the number of stem cells and by the rate of differentiation process *per se*.

In the present study, we cultured and characterized BMSC isolated from mouse. We show that this population contains MSC capable of three-lineage differentiation and colony formation in CFU-A. In order to distinguish among the effects of O_2_ on BMSC stemness and differentiation, we exposed cells at various stages of culture to different O_2_ tensions. We found that 3% O_2_ tension applied during the propagation stage selects a population of cells with stem cell characteristics and preserves their stemness. These cells undergo massive differentiation when transferred to 21% O_2_ for the differentiation stage.

The underlying mechanisms of O_2_ impact on the cellular properties are multiple. Depending on the cellular content, O_2_ either directly participates in metabolic reactions or modifies performance of other factors that are relevant for cellular stemness and differentiation, such as ROS or genetic regulators including p53 [19–21]. Here, we also show that regulation of BMSC differentiation by 3% O_2_ was realized through p53 and ROS independent pathways.

## 2. Results

### 2.1. Characterization of BMSC Cultured in 21% O_2_

**Figure 1:**
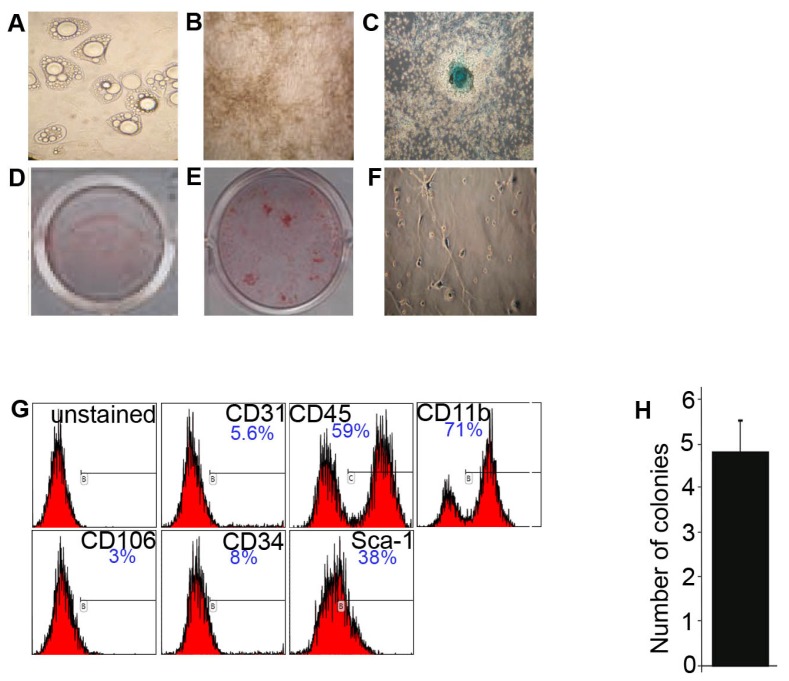
Characterization of BMSC. ***Upper panel***: BMSC are capable of multilineage differentiation. Representative views of BMSC differentiated into adipocytes (**A**), osteocytes (**B**), chondrocytes (**C**) as confirmed by Alcian Blue (**C**), Oil Red O (**D**) and Alizarin Red S (**E**) staining; and into neuron-shaped cells (**F**). ***Lower panel***: (**G**) CD profile of BMSC at day 7; (**H**) The amount of colonies detected in CFU-A per 10^5^ of seeded cells.

First, we looked at the differentiation potential of BMSC. Cells either differentiated into adipocytes or osteocytes when treated with corresponding differentiation media (Figure 1A,B), or formed spheroids of cartilage-like nature [22] upon high seeding density in chondrocitic differentiation medium (Figure 1C). Differentiation towards these lineages was confirmed by specific staining (Figure 1C–E). Upon treatment with neuronal differentiation medium, BMSC showed neuronal-like morphology (Figure 1F). Analysis of the CD profile showed that cell population at day 7 of culture contained cells expressing the hematopoietic/endothelial markers CD31, CD45, CD11b (Figure 1G). These cells became less frequent in culture with time. We also identified cells expressing CD106, CD34, and Sca-1 that are reported to be present on mMSC (Figure 1G) [7,23]. These results indicate that our BMSC preparations contained a population of MSC that was confirmed by adherence to plastic, multilineage differentiation and a set of CDs previously reported to be expressed by mMSC.

By taking the advantage of the fact that only MSC give rise to colonies when cultured at low seeding density on plastic [24], we estimated the amount of MSC in BMSC cultures by calculating the number of colonies growing in CFU-A. The results from CFU-A revealed the presence of 4.8 (±0.7) MSC per 10^5^ plated nucleated bone marrow cells (0.005%) (Figure 1H).

### 2.2. BMSC Proliferation Enhanced in 3% O_2_

**Figure 2:**
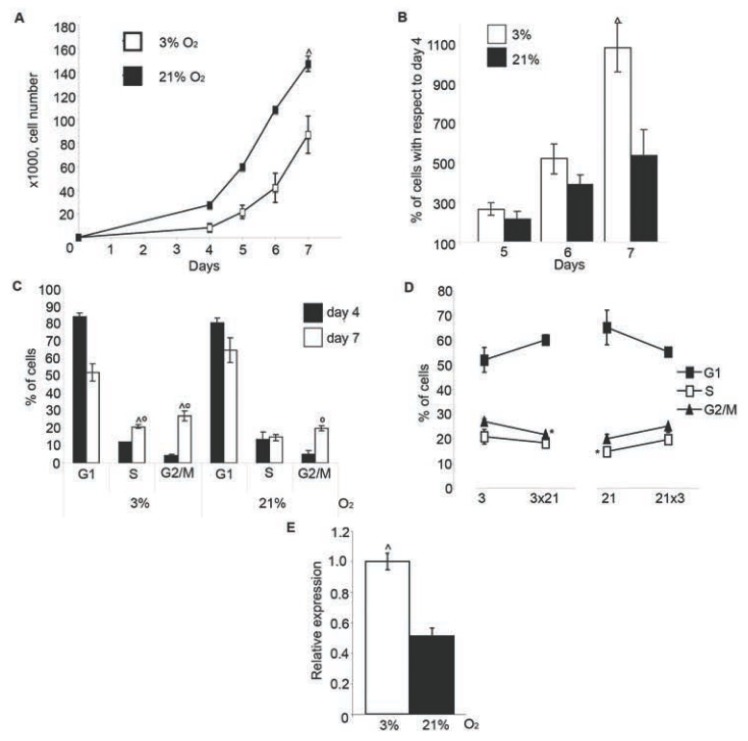
Effect of 3% O_2_ on the BMSC growth. (**A**) BMSC were seeded in quadruplicates, as described in the methods section, and cultured for seven days. Starting from day 4, cells were counted daily. Results are presented as number of cells recovered from cm_2_ of culture dish; (**B**) BMSC were cultured as in A. Number of cells at days 5, 6 and 7 are presented as percentage to day 4; (**C**) Cell cycle analysis of BMSC cultured for four (day 4) or seven (day 7) days; (**D**) Cell cycle analysis of BMSC cultured for seven days in 3% (3 × 3) or 21% O_2_ (21 × 21), or cultured for 4 days in 3% O_2_ with subsequent switch to 21% for three days (3 × 21) or *vice versa* (21 × 3); (**E**) Expression of Vegfr1 in BMSC cultured for one week as estimated by qPCR. The difference is significant at *p* < 0.05: ^ for cells cultured in 3% and 21% O_2_; ∘ for cells at day 4 and day 7 of culture; * for cells switched from 3% to 21% O_2_ and *vice versa.*

We checked if hypoxia had any effect on BMSC growth. Upon isolation from bone marrow, only few cells attached to the plastic. There was no visible increase in the amount of cells up to day 4 when rapid clonal expansion took place. At day 7, cultures reached confluence. The total number of cells recovered in 3% O_2_ at this point was reduced by almost half compared to 21% O_2_ (Figure 2A). The analysis of the cellular growth rate revealed that 3% O_2_ prolonged the lag phase of cultured cells. From day 4, the growth rate of BMSC cultured in 3% O_2_ continuously increased and on day 7 cells proliferated even faster in 3% O_2_ than in 21% O_2_ (Figure 2A,B). We confirmed this trend by cell cycle analysis. On day 4, the fraction of cells in S-G2/M phases was lower in 3% O_2_ when compared to 21% O_2_, but it significantly increased by day 7 and even exceeded the equivalent fraction in 21% O_2_ (Figure 2C). Switching the cells after growing four days at 3% O_2_ to three more days at 21% O_2_ reduced their fraction in S-G2/M. On the contrary, change from 21% to 3% O_2_ augmented the amount of cells in S-G2/M (Figure 2D).

It is known that low O_2_ induces stabilization of the transcription factor Hif-1α, which regulates cellular response to hypoxia [25]. Even though 3% O_2_ was not sufficient to trigger a measurable stabilization of the Hif-1α protein in BMSC cultures in our studies, we found that the expression of the Hif-1α responsive gene Vegfr1 increased upon exposure of cells to 3% O_2_ (Figure 2E) as previously described [26].

These data suggest that 3% O_2_ effectively triggers hypoxic response and, after a period of adaptation, stimulates the growth of BMSC.

### 2.3. Number and Proliferation of MSC Increased at 3% O_2_

**Figure 3:**
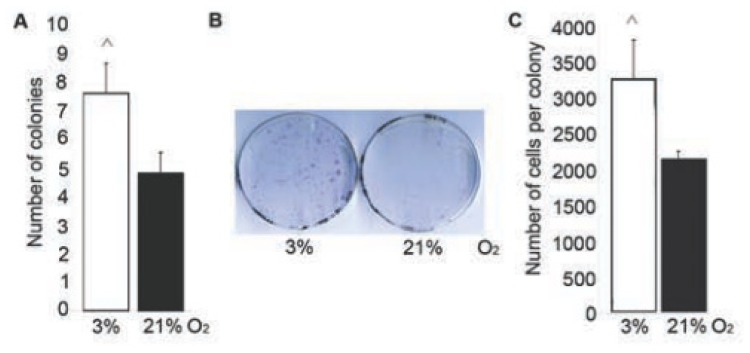
Effect of 3% O_2_ on MSC amount and proliferation. (**A**) Amount of colonies detected in CFU-A per 10^5^ of seeded cells. (**B**) Representative view of colonies in CFU-A at day 7. (**C**) Average number of cells per colony in CFU-A. The difference is significant for cells cultured in 3% and 21% O_2_: ^, *p* < 0.05.

CFU-A was performed on total BMSC to evaluate the effect of O_2_ tension on the number of MSC. Three percent of O_2_ augmented the amount of colony-forming cells by 1.6 fold in comparison with 21% O_2_ (Figure 3A,B). In addition, the size of colonies and the average number of cells per colony increased in 3% O_2_ (Figure 3B,C).

Obtained results suggest that 3% O_2_ enhances the yield and proliferation of MSC contributing to the increased growth rate of the total BMSC population.

### 2.4. 3% O_2_ Inhibited Differentiation of BMSC

**Figure 4:**
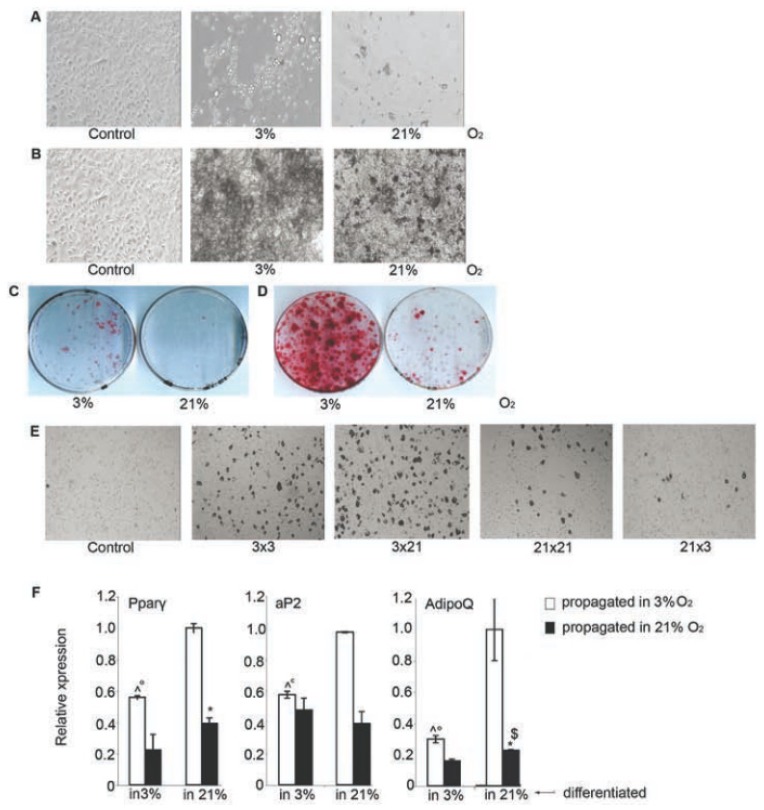
Effect of 3% O_2_ on BMSC differentiation. Pictures of BMSC differentiated into adipocytes (**A**) or osteocytes (**B**); Differentiation of BMSC into adipocytes (**C**) and osteocytes (**D**) in CFU-A; (**E**) BMSC were both propagated and differentiated in 3% O_2_ (3 × 3) or 21% O_2_ (21 × 21); or propagated in 3% O_2_ and differentiated in 21% O_2_ (3 × 21) or *vice versa* (21 × 3). Representative picture of cells not stimulated with differentiation media is shown as control; (**F**) Relative expression of Pparγ, aP2, adipoQ in cultures differentiated as in E as estimated by qPCR. Expression of these genes was insignificant in cultures untreated with differentiation media. The difference is statistically significant at *p* < 0.05 for cells: ° differentiated in 3% O_2_ upon propagation in 3% or 21% O_2_, * differentiated in 21% O_2_ upon propagation in 3% or 21% O_2_, ^ differentiated in 3% or 21% O_2_ upon propagation in 3% O_2_, $ differentiated in 3% or 21% O_2_ upon propagation in 21% O_2_.

Next, we tested the differentiation potential of BMSC in 3% and 21% O_2_. In agreement with previously reported data, few adipocyte-like cells, with accumulated lipid droplets and characteristic round adipocyte morphology, were detected when BMSC were both propagated and differentiated in 21% O_2_ [8] (Figure 4A). The frequency of adipocytes remarkably increased if both these steps were performed in 3% O_2_ (Figure 4A).

Osteocytic differentiation was also more efficient when BMSC were propagated and differentiated in 3% O_2_. BMSC cultured in 3% O_2_ produced higher amount of mineralized extracellular matrix in comparison with cells cultured in 21% O_2_ (Figure 4B).

We observed the similar pattern of differentiation in CFU-A, where 3% O_2_ augmented both the amount of colonies undergoing adipocytic and osteocytic differentiation and the efficiency of their differentiation (Figure 4C,D).

Three percent of O_2_ may affect the outcome of BMSC differentiation by regulating intrinsic differentiation processes or by selecting cells capable of differentiation (stem cells). To address this issue, BMSC initially amplified at 3% O_2_ were induced to differentiate at 21% O_2_. This culture scheme further promoted the outcome of BMSC differentiation in comparison with cells cultured at 3% O_2_ only (Figure 4E). In contrast, when cells were amplified in 21% O_2_ and switched for differentiation to 3% O_2_, the differentiation yield was drastically reduced, almost abolished. We confirmed this pattern of BMSC adipocytic differentiation by qPCR, which showed the highest expression of adipocytic differentiation markers Pparγ2 [27], aP2 [28] and adipoQ [29] in cells amplified in 3% O_2_ and switched for the differentiation to 21% O_2_ (Figure 4F).

Thus 3% O_2_ inhibits the differentiation process *per se.*

### 2.5. 3% O_2_ Enhanced Pluripotency Markers Expression in BMSC

Since cells amplified in 3% O_2_ subsequently differentiated more efficiently, we hypothesized that 3% O_2_ selects for stem cells. Therefore, we checked whether 3% O_2_ affected the stemness of BMSC cultures.

Oct-4 and Rex-1 are pluripotent stem cell markers, which were initially described in embryonic stem cells [30,31]. Induction of their expression by low O_2_ is observed in various types of cells [32] and is associated with increased stemness in hMSC [10,13].

**Figure 5:**
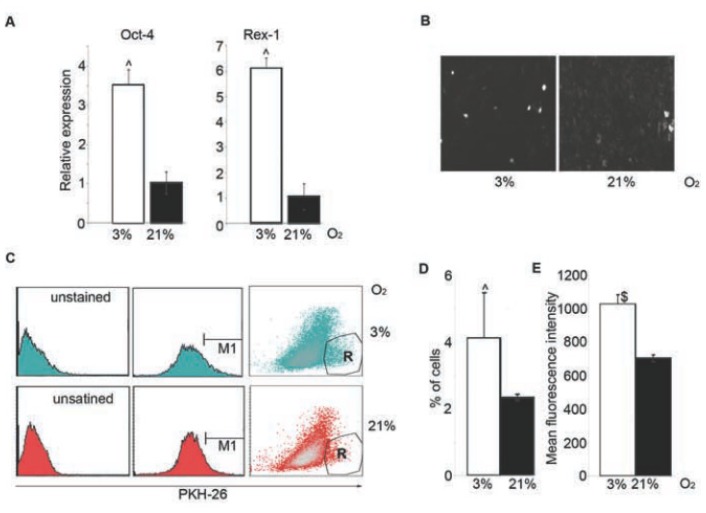
Effect of 3% O_2_ on BMSC stem cell markers expression and amount of quiescent cells. (**A**) Expression of Oct-4 and Rex-1 in BMSC at day 7; (**B**) Pictures of BMSC observed with fluorescent microscope upon PKH-26 staining; (**C**) FACS histograms of BMSC upon PKH-26 staining. R region (=M1 region) represents cells with the highest PKH-26 fluorescence; (**D**) Percentage of cells included in R region from C; (**E**) Mean fluorescence intensity of cells gated by R region. The difference is statistically significant for cells cultured in 3% and 21% O_2_: ^, *p* < 0.05, $, *p* < 0.005.

Our qPCR results revealed that 3% O_2_ enhanced expression of Rex-1 and Oct-4 in BMSC (Figure 5A).

### 2.6. 3% O_2_ Decreases the Cycling Fraction of BMSC

Adult stem cells divide occasionally [33] to renew themselves and generate progeny committed to differentiation [34]. Thus, the amount of rarely dividing cells correlates with the amount of stem cells. We estimated the amount of quiescent BMSC under different O_2_ conditions by staining the culture with the vital fluorescent dye PKH-26 that stably integrates into the plasma membrane. When labeled cells divide, daughter cells receive half of the dye, hence PKH-26 signal decreases upon proliferation. In our experiments, both the number of PKH-26 positive cells and their fluorescence intensity were increased in BMSC propagated in 3% O_2_ in comparison with control cells cultured in 21% O_2_ (Figure 5B–E).

### 2.7. 3% O_2_ Affected BMSC Differentiation through ROS and p53 Independent Mechanisms

**Figure 6:**
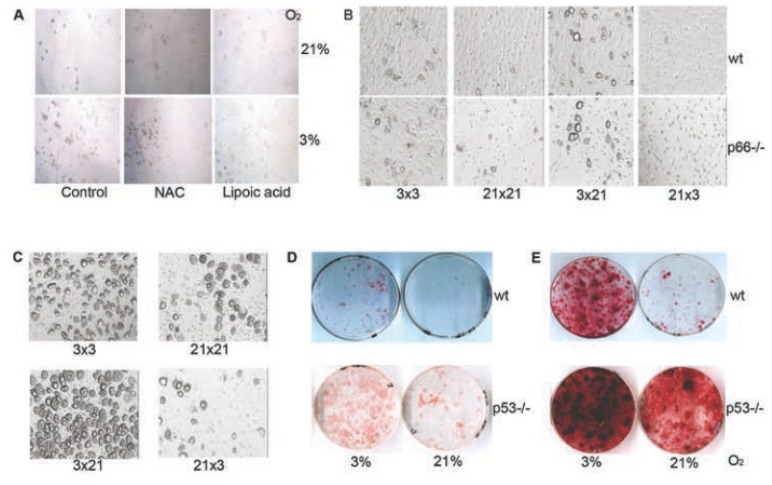
Effect of 3% O_2_ on BMSC differentiation is independent of ROS and p53. (**A**) Representative pictures of BMSC cultured and differentiated in 3% or 21% O_2_ in standard adipocyte differentiation media (control) or supplemented with NAC or lipoic acid; (**B**) Representative pictures of BMSC isolated from wild type (wt) and p66^Shc−/−^ mice and propagated and differentiated into adipocytes in 3% (3 × 3) or 21% O_2_ (21 × 21); or propagated in 3% O_2_ and differentiated in 21% O_2_ (3 × 21), or *vice versa* (21 × 3); (**C**) Representative images of p53^−/−^ BMSC propagated and differentiated in 3% (3 × 3) or 21% O_2_ (21 × 21); or amplified in 3% O_2_ and differentiated in 21% O_2_ (3 × 21), or *vice versa* (21 × 3); (**D**) Oil Red O staining of wt and p53^−/−^ BMSC stimulated to adipocytic differentiation in CFU-A; (**E**) Alizarin Red S staining of wt and p53^−/−^ BMSC stimulated to osteocytic differentiation in CFU-A.

To validate the role of ROS in O_2_’s effect on the stemness and differentiation, we tested differentiation of BMSC treated with antioxidants such as lipoic acid or *N*-Acetylcysteine (NAC), or differentiation of BMSC derived from p66^Shc^-deficient mice characterized by reduced ROS levels ([35] and Figure S1). Results revealed that reducing ROS levels by chemicals or by genetic means had no effect on the ability of BMSC to differentiate under various O_2_ levels (Figure 6A,B).

Next, we checked if low O_2_ tension regulated BMSC differentiation in a p53 dependent manner. To this aim, we investigated differentiation of BMSC from p53 deficient mice in 3% and 21% O_2_. P53^−/−^ BMSC showed significantly higher differentiation ability. Nevertheless, the pattern of BMSC differentiation under various O_2_ tensions remained unchanged (Figure 6C–E). It appears that the O_2_ availability modulates BMSC differentiation independently from ROS and p53.

## 3. Discussion

O_2_ availability changes among tissues and within the same tissue depending on surrounding capillaries. Altered O_2_ distribution is characteristic of several pathological conditions, such as ischemia [36], inflammation [37], wound injury, diabetes, and cancer [38]. Due to the delicate balance between the need of O_2_ for energetic metabolism, and the oxidative damage induced by excessive exposure to O_2_, changes in O_2_ levels represent a serious challenge for every cell type. Activation of specific O_2_ sensitive pathways is particularly important for cells migrating into different sites of the organism, such as stem cells from bone marrow [39,40]. In these cells, O_2_ levels not only activate stress adaptive responses, but also regulate recruitment and further differentiation, as observed during systemic hypoxia in high quote [1,41] or hyperoxia in hyperbaric chamber [42].

We investigated the effect of O_2_ variations on BMSC properties. After an initial period of adaptation to hypoxic conditions, the proliferation rate of BMSC was higher in 3% O_2_ than in 21% O_2_. Three percent of O_2_ also increased the number of colonies formed in CFU-A, the expression of pluripotency markers, and the amount of quiescent cells, thus suggesting that low O_2_ selects for MSC. The differentiation of cells both propagated and differentiated in 3% O_2_ was more efficient in comparison with 21% O_2_. However, the differentiation yield was further increased upon switching of cells from 3% to 21% O_2_ for the differentiation stage, whereas it was remarkably suppressed if cells were switched from 21% to 3% O_2_. It appears, therefore, that the differentiation process *per se* is inhibited in low O_2_, whereas it is strongly boosted by increasing O_2_ tension.

Our findings indicate that BMSC cultured in 3% O_2_ were enriched in MSC by corresponding to the criteria of adherence to plastic, multilineage differentiation and a characterized CD profile. As a consequence of such increased the amount of MSC, differentiation outcome was increased, whereas the differentiation process *per se* was inhibited in reality.

At molecular level, we exclude such critical regulators of cellular stemness and differentiation as p53 and ROS from the transmitting O_2_ effects on MSC, as neither chemical antioxidant nor genetic mutation that reduces ROS concentration or p53 deletion affected O_2_ regulation of BMSC differentiation. Hif-1α, a major factor involved in O_2_ sensing, is also apparently not involved. Even though 3% O_2_ triggered the expression of Hif-1α inducible gene Vegfr1, O_2_ oscillations were not sufficient to induce detectable Hif-1α stability, and knocking down its expression by RNAi did not alter O_2_ effects (data not shown).

It seems therefore, that a particular O_2_-sensing mechanism controls MSC stemness/differentiation response.

Finally, despite being far from the understanding of the molecular basis underlying O_2_ effects on MSC, fine-tuning of O_2_ tension to the specific stages of *in vitro* culture may represent an important factor to obtain high-quality MSC for the needs of regenerative therapy.

## 4. Materials and Methods

### 4.1. Reagents

If not stated otherwise, all reagents were purchased from Sigma (St. Louis, MO, USA).

### 4.2. BMSC Isolation and Amplification

Wild type, p53^−/−^ or p66^Shc−/−^ 8–10 week-old male mice in C57Bl/6 background were sacrificed by cervical dislocation, and tibia and femurs were collected. Bone marrow was flushed out with growth medium (DMEM-HG supplemented with 20% FBS, 2 mM glutamine, 100 U/mL penicillin, 100 μg/mL streptomycin, 20 mM HEPES pH 7.4) and passed through 27 G needle. The obtained suspension was centrifuged at 300× *g* for 10 min. Pellet was resuspended in growth medium and cells were seeded at a density of 2 × 10^5^ nucleated cells/cm^2^ (day 0) and left undisturbed for 72 h. Afterwards, the medium was changed every 48 h in growing cultures and twice per week in differentiating cultures. At day 7, cells were split at a density of 10^6^/cm^2^ and cultured in DMEM-HG supplemented with 10% FBS, 2 mM glutamine, 100 U/mL penicillin and 100 μg/mL streptomycin. The next day, differentiation was induced. Cultures were kept at 37 °C in a gas mixture containing 3% or 21% O_2_, 5% CO_2_ and balanced with N_2_.

All the *in vivo* experiments were performed in accordance with Italian laws and regulations.

### 4.3. Differentiation

For adipocytic differentiation cells were cultured in adipocytic differentiation medium (DMEM-HG and Ham F12 mixed 3:2, supplemented with 10% horse serum, 2 mM glutamine, 100 U/mL penicillin, 100 μg/mL streptomycin, 100 nM dexamethasone, 5 μg/mL insulin, and 10 mM nicotinamide) for 14 days. For certain experiments the differentiation medium was supplemented with 250 μM lipoic acid or 500 μM *N*-acetylcystein (NAC). Oil Red O staining was performed as described by others [43].

For osteogenic differentiation, cells were cultured in osteogenic differentiation medium (DMEM-HG supplemented with 10% FBS, 2 mM glutamine, 100 U/mL penicillin, 100 μg/mL streptomycin, 50 μM ascorbic acid, 10 mM beta-glycerol-3-phosphate, and 100 nM dexamethasone) for 14 days. For Alizarin Red S staining, cells were fixed in formalin, washed with water, incubated for 5 min with 2% Alizarin Red solution, pH 4.1 and washed twice with water.

For chondrogenic differentiation a method of spheroids culture was used [22]. For Alcian Blue staining, obtained spheroids were fixed in 10% formalin and overloaded with 1% Alcian Blue-8GX solution overnight.

For neuronal differentiation, cells were split every other day, starting from passage 1 in growth medium containing 10 ng/mL bFGF. At passage 5, neuronal differentiation medium (alpha-MEM, supplemented with 0.1% of FBS, 1% DMSO, 10 μM forskolin, 1 μM hydrocortisone, 5 μg/mL insulin, 10 ng/mL bFGF) was applied for 12–24 h.

### 4.4. CFU-A

For CFU-A cells isolated from bone marrow (passage 0) were seeded at a density of 50 × 10^3^ nucleated cells/cm^2^. At day 7, cells were washed with PBS, fixed in 10% formalin, incubated with Giemsa dye for 10 min and washed three times with water.

Differentiation of cultures seeded in CFU-A was induced at day 8, as described above, but without split of the cells.

### 4.5. PKH-26 Staining

Staining was performed with PKH-26 Red Fluorescent Cell Linker Mini Kit. At day 6 cells were trypsinized and collected by centrifugation. Pellet was resuspended in Diluent C, mixed with 4 × 10^−6^ M PKH-26 solution and incubated for 2 min. The reaction was terminated by the addition of an equal FBS volume for 1 min. Next, an equal volume of complete medium was added and the suspension was centrifuged. Afterwards cells were washed three times with PBS at 400× *g* for 5 min and seeded. Fluorescence of cultures was observed after 48 h with a fluorescent microscope (Olympus BX 61) or analyzed by FACS Callibur Cytometry system.

### 4.6. Total RNA Extraction

Total RNA extraction was performed using the RNeasy isolation kit (Qiagen, Hilden, Germany).

### 4.7. CDNA Synthesis and qPCR

RNAs were reverse transcribed using SuperScript II reverse transcriptase and random primers (Life Technologies, Carlsbad, CA, USA). Obtained cDNA was used for determination of relative levels of specific mRNA with a 5′ nuclease assay (TaqMan) chemistry system. Gapdh expression was used for normalization. QPCR was performed on an ABI 7900HT sequence detection system. Primer sequences are listed in Table S1.

### 4.8. CD Profiling

For flow cytometry analysis, labeling of cells was performed with the following antibodies: fluorescein isothiocyanate-conjugated anti-CD31, anti-CD34, anti-CD106 (eBioscience); phycoerythrin-conjugated anti-Sca-1, anti-CD45 (BD Pharmingen), anti-CD44 (eBioscience), and biotin-conjugated anti-CD11b (BD Pharmingen), all according to manufacturer’s instructions.

### 4.9. Cell Cycle Analysis

For cell cycle analysis, cells were resuspended in ice-cold 70% ethanol/PBS, incubated for 30 min on ice, washed in 1% BSA/PBS, and incubated for 1 h in 250 μg/mL RNase/50 μg/mL propidium iodide solution and analyzed by using Cell Quest software (Beckman Ltd., Brea, CA, USA).

### 4.10. ROS Analysis

Cells were stained with 70 μM DCFDA and analyzed by FACS as described [19].

### 4.11. Statistical Analysis

Experiments were repeated a minimum of three times, with the exception of CD profile analysis, which was performed twice. Data are presented as means ± standard deviation and were analyzed by Student’s *t* test. Differences between means were assessed by one-way analysis of variance. The minimum level of significance was set at *p* < 0.05.

## Supplementary Information



## Abbreviations

BMSC: bone marrow stromal cells
CFU-A: colony-forming unit assay
MSC: mesenchymal stem cells
NAC: *N*-acetylcysteine
ROS: reactive oxygen species
