# Supplementary material for: Low Oxygen Tension Maintains Multipotency, Whereas Normoxia Increases Differentiation of Mouse Bone Marrow Stromal Cells
Source: Int J Mol Sci. 2013 Jan 22;14(1):2119–34. doi: 10.3390/ijms14012119 (PMC3565369; doi:10.3390/ijms14012119)
Supplement: Supplementary file 1 [file ijms-14-02119-s001.pdf]

Supplementary Information

Table S1. Primers used in qPCR.

Rex1	F: GCGGTGTGTACTGTGGTGTCTT	R: TCTTGCACCCGGCTTGA
Oct4	F: GTCCGCCCGCATACGA	R: AGTCCAACCTGAGGTCCACAGT
Gapdh	F: GCACCGTCAAGGCTGAGAAC	R: AGGGATCTCGCTCCTGGAA
Pparg	F: GCCCACCAACTTCGGAATC	R: TGCGAGTGGTCTTCCATCAC
aP2	F: CCGCAGACGACAGGAAGGT	R: AGGGCCCCGCCATCT
adipoQ	F: AACCCCTGGCAGGAAAGG	R: TGAACGCTGAGCGATACACAT

Figure S1. Intracellular ROS levels in wt and $p66^{Shc-/-}$ BMSC.

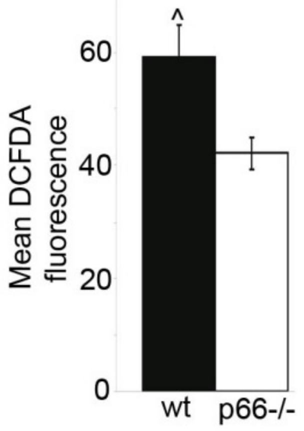

© 2013 by the authors; licensee MDPI, Basel, Switzerland. This article is an open access article distributed under the terms and conditions of the Creative Commons Attribution license (http://creativecommons.org/licenses/by/3.0/).
